# Human Telomere Length Correlates to the Size of the Associated Chromosome Arm

**DOI:** 10.1371/journal.pone.0006013

**Published:** 2009-06-23

**Authors:** Jasen L. Wise, Richard J. Crout, Daniel W. McNeil, Robert J. Weyant, Mary L. Marazita, Sharon L. Wenger

**Affiliations:** 1 Department of Pathology, West Virginia University, Morgantown, West Virginia, United States of America; 2 School of Dentistry, West Virginia University, Morgantown, West Virginia, United States of America; 3 Department of Dental Public Health and Information Management, University of Pittsburgh School of Dental Medicine, Pittsburgh, Pennsylvania, United States of America; 4 Center for Craniofacial and Dental Genetics, Departments of Oral Biology and Human Genetics, University of Pittsburgh, Pittsburgh, Pennsylvania, United States of America; Duke University, United States of America

## Abstract

The majority of human telomere length studies have focused on the overall length of telomeres within a cell. In fact, very few studies have examined telomere length for individual chromosome arms. The objective of this study was to examine the relationship between chromosome arm size and the relative length of the associated telomere. Quantitative Fluorescence *In Situ* Hybridization (Q-FISH) was used to measure the relative telomere length of each chromosome arm in metaphases from cultured lymphocytes of 17 individuals. A statistically significant positive correlation (r = 0.6) was found between telomere length and the size of the associated chromosome arm, which was estimated based on megabase pair measurements from http://www.ncbi.nlm.nih.gov/projects/mapview/.

## Introduction

The human telomere is a highly repetitive GC rich protective structure located at the terminal regions of the chromosomes. The telomere “caps” the ends of chromosomes to prevent end-to-end fusions, which could result in structural and segmental aneuploidy. The inverse relationships between overall telomere length and its role in aging and premature aging diseases have been well documented [1]–[3]. However, only a few studies have examined the length of telomeres for individual chromosome arms in humans.

Lansdorp and colleagues [4] had examined the telomere length of human chromosomes in cells from different tissues and found that sister chromatid telomere length was similar; however, the distribution of telomere length between chromosomes was not random. Martens and colleagues [5] found that there was a significant difference between the telomere length of individual chromosome arms, with a weak positive correlation between the length of chromosome arms and the corresponding telomeres. Graakjaer and colleagues [6]–[8] had found that human telomere length correlated well with chromosome length but did not have as strong a correlation with individual chromosome arm size.

The purpose of this study was to examine telomere length of individual chromosome arms in metaphase cells from cultured lymphocytes of 17 individuals using quantitative fluorescence *in situ* hybridization (Q-FISH). Q-FISH for telomere length was performed using peptide nucleic acid (PNA) probes which are highly specific and provide a high resolution (200 bp) [9] allowing for quantitative measurement of telomeres by digitally measuring the intensity of probe signals on metaphase chromosomes against a reference probe. Rather than rely on the photographic size of each chromosome arm, the length of chromosome arms in Mb was obtained from the National Center for Biotechnology Information (NCBI) map viewer website.

## Methods

Subjects for this study were drawn from the Center for Oral Health Research in Appalachia (COHRA) [10], an ongoing cross-sectional oral health etiology study. COHRA ascertains families from two central West Virginia counties and two western Pennsylvania counties and performs a detailed assessment protocol after written informed consent. The institutional review boards of the University of Pittsburgh and West Virginia University approved the consent process. Seventeen COHRA subjects were included in the current study: 8 male and 9 female, with ages ranging from 2 to 45 years of age, average age 18.9 years, and median age 15 years (Table 1).

**Table 1:** A bivariate fit of average telomere length by chromosome size (in Mb from table 2) was performed to correlate the relationship between physical chromosome size and relative telomere length for each individual in the study.

Individual	Sex	Age	p =
1	Female	7	0.0107*
2	Female	15	0.006*
3	Female	9	0.4061
4	Female	9	0.0575
5	Female	5	0.0008*
6	Female	6	0.0686
7	Male	45	0.0338*
8	Male	39	0.3145
9	Male	34	0.019*
10	Male	35	0.1919
11	Female	36	0.0094*
12	Female	28	<0.0001*
13	Female	33	0.0252*
14	Male	12	0.0086*
15	Male	6	0.0506
16	Male	15	0.0161*
17	Male	4	0.0259*
Overall			<0.0001*
			*p<0.05

A positive correlation was found for all individuals; statistically significant correlations were seen in 11 of 17 (65%) and another 3 of 17 were close to the 0.05 cutoff for significance.

One ml of blood was added to 7 ml of culture media in a 15 ml sterile conical centrifuge tube and mixed by inversion. The culture was incubated for 72 hours at 37°C. Ninety µl of colcemid (Gibco Cat #757575) was added to each culture for 30 minutes. Cultures were then centrifuged at 1200 rpm for 10 minutes. Supernatant was removed by aspiration and 9 ml of hypotonic solution (0.075 M KCl) was added, mixed by inversion, and incubated at 37°C for 10 minutes. Following incubation, 3 ml of fixative (1∶3 glacial acidic acid to absolute methanol) was added to each culture, mixed by inversion and centrifuged at 1200 rpm for 10 minutes. Supernatant was removed by aspiration and the cell pellet was resuspended. Fixative washes were repeated until the cell pellet was clean and white. The cultures were then stored at 4°C. At room temperature the cell pellet was re-suspended to an appropriate volume to obtain 5 or more metaphase cells per 10× field of view.

Cells were dropped onto slides, which were then immersed for: 2 min. in Tris Buffered Saline (TBS), 2 min. in 3.7% formaldehyde in 1× TBS, 10 min. in TBS, 10 min. in Pre-Treatment solution (DAKO, Glostrup, Denmark) and 10 min. in TBS. The slides were immersed through a series of ethanol washes of 70%, 85%, and 100% for 1 min. each and then allowed to air dry at room temperature.

Hybridization protocol was followed as provided by the manufacturer (DAKO). Ten µl of FITC PNA telomere probe mix (DAKO) and FITC PNA chromosome 2 centromere probe (courtesy of DAKO) was added to prepared slides, coverslipped and sealed with tape. The probe and chromosome preparation were codenatured for 5 min. at 80°C and placed in the dark at room temperature for 30 min. Slides were immersed in the supplied rinse solution (DAKO) for 1 min. The coverslips were removed and the slides were washed in the supplied wash solution for 5 min. at 65°C. The slides were then dehydrated by immersion through a series of ethanol washes of 70%, 85%, and 100% for 1 min. each and allowed to air dry at room temperature. Twenty µl of 1× DAPI counterstain (In Situs, Albuquerque, NM) was applied to the dried slides and coverslipped (20 mm×50 mm).

**Figure 1:**
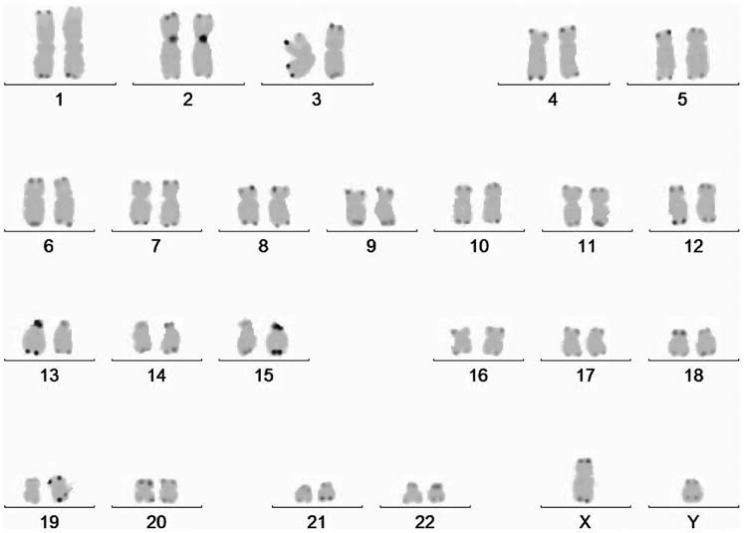
Grayscale image of a karyogram showing probe fluorescence at each telomere and the centromere reference probe on chromosome 2 using PNA FISH probes.

Metaphases were photographed using ISIS (MetaSystems, Altlussheim, Germany) (Fig. 1) software on a Leica epi-fluorescent microscope equipped with a DAPI single bandpass, and a FITC single bandpass filter. The images were then background corrected, and the color channels were placed in inverted grayscale mode, approximating a G-banding pattern to allow for easier karyotyping. Telomere lengths were measured in 76 metaphase cells from 17 individuals (8 male and 9 female), giving 152 individual measurements for each chromosome arm (with the exception of the X (n = 120) and Y (n = 32) chromosomes).

The telomere measurement software package from MetaSystems was used to quantify telomere length on the p and q arms of each chromosome relative to the reference signal on chromosome 2. Our measurements were reported as arbitrary units of Relative Telomere Length Units (RTLU), which is the ratio of telomere signal intensity to the centromere 2 reference signal. Pixel intensity measurements do not have the resolution to translate into megabase pairs. The size of each chromosome arm (in Mb) was obtained from data provided online from the National Center for Biotechnology Information (NCBI) Map Viewer [11]. The RTLU was compared to chromosome arm size as measured in Mb for individual subjects as well as pooled data. All statistical analysis was performed using JMP statistical software.

## Results

Telomere length as RTLU and chromosome arm length in Mb was compared for each individual in our study to determine if the pattern profile showed consistency between individuals (Table 1). Direct correlation was found for all individuals; statistically significant correlations were seen in 11 of 17 (65%). Half of the remaining were close to the 0.05 cutoff for significance. By using Mb pair values in our calculations of chromosome arm length, the variability of chromosome arm measurement due to varying degrees of chromosome condensation could be avoided.

**Figure 2:**
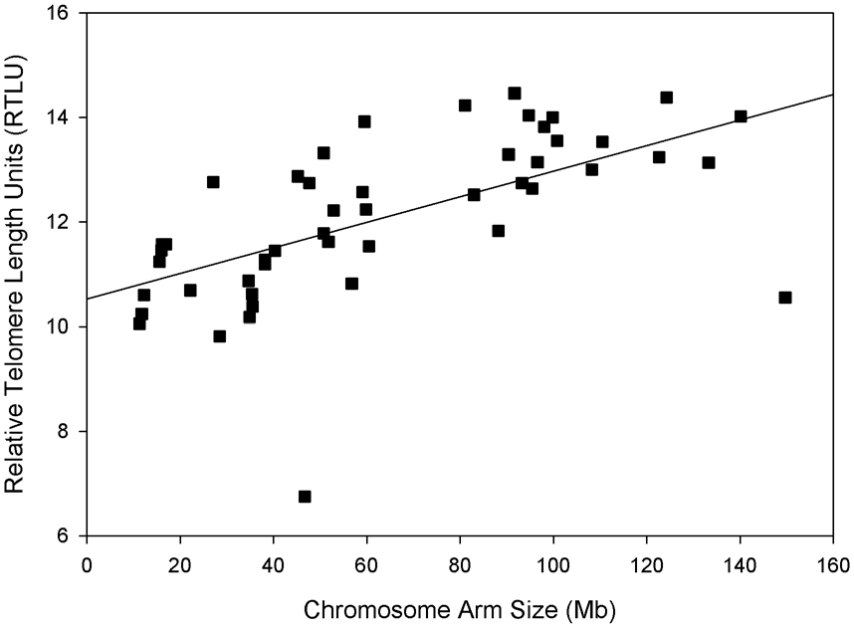
Chromosome arm sizes measured in Mb pairs are plotted against relative telomere length units (RTLU) from data presented in Table 2. The linear regression line corresponds to a correlation coefficient of 0.6 with a significant positive correlation (p<0.0001).

Due to consistency of the relationship between lengths for telomeres and chromosome arm relationships among individuals, the data were pooled for each chromosome arm to determine the mean value for each corresponding telomere ratio to the centromere signal, RTLU (Table 2). After pooling of data, average telomere length (RTLU) was compared to chromosome arm size (in Mb), which had a highly significant (p<0.0001) direct positive correlation (Fig. 2) using bivariate analysis with a correlation coefficient of 0.60.

**Table 2:** Chromosome arm size in Mb, obtained from the NCBI map viewer website and the corresponding RTLU from data pooled for all individuals from table 1.

Chromosome Arm	Chromosome Arm Size (M bp)	Relative Telomere Length Units
2q	149.7	10.55
4q	140.2	14.02
5q	133.3	13.13
1p	124.3	14.38
1q	122.7	13.24
6q	110.5	13.53
3q	108.3	13.00
8q	100.8	13.55
7q	99.9	14.00
13q	98.0	13.82
12q	96.6	13.14
Xq	95.5	12.64
10q	94.7	14.04
2p	93.3	12.74
3p	91.7	14.46
14q	90.4	13.29
9q	88.2	11.83
15q	83.0	12.52
11q	81.1	14.23
6p	60.5	11.53
18q	59.9	12.24
Xp	59.5	13.92
7p	59.1	12.57
17q	56.8	10.82
11p	52.9	12.22
9p	51.8	11.62
16q	50.8	11.78
4p	50.8	13.32
5p	47.7	12.74
Yq	46.7	6.75
8p	45.2	12.87
10p	40.3	11.45
16p	38.2	11.28
22q	38.2	11.19
19q	35.5	10.38
12p	35.4	10.62
20q	34.9	10.18
21q	34.7	10.87
19p	28.5	9.82
20p	27.1	12.76
17p	22.2	10.69
15p	17.0	11.57
18p	16.1	11.57
13p	16.0	11.46
14p	15.6	11.24
21p	12.3	10.60
22p	11.8	10.24
Yp	11.3	10.06

## Discussion

In this study, the human telomere length for each chromosome arm was examined to help clarify the relationship of telomere length and chromosome arm size, since few studies have examined this relationship in detail. Our data shows that telomere length directly correlates to the length of the chromosome arm.

Other studies have examined the telomere length profile in humans with regard to chromosome arm size and telomere length. Martens and colleagues [5] found a weak positive correlation between human chromosome arm size and corresponding telomere length; however, they did not show their data and only briefly commented on the findings. Graakjaer and colleagues [6]–[8], [11] found positive correlations (correlation coefficient = 0.51 and 0.79, respectively) between human chromosome arm size and corresponding telomere length from lymphocytes. Our overall data had a correlation coefficient of 0.60.

Several studies have found statistically significant correlations of telomere length to the total size of each chromosome (summation of p and q arms) [5]–[8], [12]. We observed a similar correlation with our data (not shown) (p<0.0001). The correlations of telomere length with chromosome arm size and total chromosome size would be expected since the summation of p and q chromosome arm size and the summation of p and q telomere length would show the same proportional relationships as single arm and single telomere measurements. Because of this property, the correlation with the smaller part suggests that individual telomere length to chromosome arm size is the functional relationship, but does not exclude the possibility of a combination of effects from both relationships. It is also important to keep in mind that correlation does not necessarily indicate causation.

Non-human studies using mouse models [13], Chinese hamster models [14], and plant *Pennisetum glaucum* (L.) (pear millet) [15] have demonstrated correlation between telomere length and chromosome arm size relationships. These findings are important because, along with human studies, they demonstrate that chromosome arm size and telomere length relationships have been conserved by evolution. Evolutionary conservation may suggest a greater functional relationship rather than structural stability. To further understand telomere-chromosome relationships it may be beneficial to examine telomere length in diverse taxonomic classes as well as examining other factors such as chromosome numbers and genomic organization.

In conclusion, the linear relationship between telomere length and physical chromosome arm size is consistent from individual to individual regardless of sex or age. Variations based on age and gender occurs but the general trend of increasing telomere length with increasing chromosome arm size remains. Our data shows that the distribution of telomere length correlates with length of chromosome arm, and suggests that the common telomere pattern in humans may be more dependent on chromosome arm size rather than the physical size of the entire chromosome.
